# Thyroseq V3 Molecular Profiling for Tailoring the Surgical Management of Hürthle Cell Neoplasms

**DOI:** 10.1155/2018/9329035

**Published:** 2018-07-16

**Authors:** Sarah Pearlstein, Arash H. Lahouti, Elana Opher, Yuri E. Nikiforov, Daniel B. Kuriloff

**Affiliations:** ^1^Department of Surgery, Lenox Hill Hospital, New York, NY, USA; ^2^Department of Pathology, Lenox Hill Hospital, New York, NY, USA; ^3^Department of Pathology, University of Pittsburgh Medical Center, Pittsburgh, PA, USA; ^4^New York Head & Neck Institute, Lenox Hill Hospital, New York, NY, USA; ^5^Zucker School of Medicine at Hofstra/Northwell, New York, NY, USA

## Abstract

Hürthle cell predominant thyroid nodules often confound the diagnostic utility of fine needle aspiration biopsy (FNAB) with cytology often interpreted as a Hürthle cell lesion with an indeterminate risk of malignancy, Bethesda category (BC) III or IV. Molecular diagnostics for Hürthle cell predominant nodules has also been disappointing in further defining the risk of malignancy. We present a case of a slowly enlarging nodule within a goiter initially reported as benign on FNAB, BC II but on subsequent FNAB suspicious for a Hürthle cell neoplasm, BC IV. The patient had initially requested a diagnostic lobectomy for a definitive diagnosis despite a higher risk of malignancy based on the size of the nodule > 4 cm alone. To better tailor this patient's treatment plan, a newer expanded gene mutation panel, ThyroSeq® v3 that includes copy number alterations (CNAs) and was recently found to have greater positive predictive value (PPV) for identifying Hürthle cell carcinoma (HCC), was performed on the FNAB material. Molecular profiling with ThyroSeq® v3 was able to predict a greater risk of carcinoma, making a more convincing argument in favor of total thyroidectomy. Surgical pathology confirmed a Hürthle cell carcinoma with 5 foci of angioinvasion and foci of capsular invasion.

## 1. Introduction

Thyroid nodules with a predominance of Hürthle cells often confound the diagnostic utility of fine needle aspiration biopsy with cytology often interpreted as a Hürthle cell lesion with an indeterminate risk of malignancy, Bethesda category III or IV. Molecular diagnostics for Hürthle cell predominant thyroid nodules, with the exception of medullary thyroid carcinoma, has also been disappointing in further defining the risk of malignancy. This diagnostic challenge occurs because Hürthle cells or oncocytic metaplasia is associated with benign nodules (cell-mediated autoimmune thyroiditis, humoral-mediated Graves' disease, and hyperplastic nodules in multinodular goiters (MNG)). Hürthle cells also occur in neoplastic conditions such as Hürthle cell adenoma, Hürthle cell carcinoma, and the oncocytic variant of papillary thyroid carcinoma. Medullary carcinoma, a C-cell derived neoplasm, can also exhibit an oncocytic appearance and is included in the differential diagnosis of Hürthle cell lesions.

Furthermore, different areas within the same nodule may yield very different degrees of Hürthle cell differentiation further confusing the cytologic interpretation. There are additional challenges; a benign Hürthle cell adenoma cannot be distinguished from a HCC without demonstrating either capsular or vascular invasion found after surgical removal on careful histopathologic assessment at multiple levels. The biological behavior of HCC varies and can present either as a minimally invasive or as a widely invasive tumor. Hürthle cell carcinoma may have a more aggressive biological behavior compared with the other well-differentiated thyroid cancers and is associated with a higher rate of distant metastases. Hürthle cell carcinoma often has less radioiodine avidity compared with other well-differentiated thyroid cancers, mandating a more complete thyroidectomy, especially for optimal adjuvant therapy for a subset of tumors with some RAI avidity in the setting of locally aggressive HCC, regional lymph node involvement, or distant metastases [1].

We present a case of a slowly enlarging nodule within a MNG initially reported as benign on FNA cytology BC II but on subsequent FNA cytology interpreted as a Hürthle cell neoplasm or suspicious for a Hürthle cell neoplasm, BC IV. Molecular profiling using ThyroSeq® v2 next-generation gene sequencing [2] revealed an absence of gene mutations or fusions but strong overexpression of the MET gene. Since this finding alone could not reliably predict a HCC, the patient had initially requested a diagnostic lobectomy for a definitive pathologic diagnosis despite a higher risk of malignancy based on the size of the nodule > 4 cm alone. To better tailor this patient's treatment plan, the ThyroSeq® v3 panel, recently found to have greater positive predictive value (PPV) for identifying Hürthle cell malignancies, was performed on the FNA material. Molecular profiling with ThyroSeq® v3 was able to predict a greater risk of HCC, making a more convincing argument in favor of total thyroidectomy. This case report illustrates the important role of molecular diagnostics, specifically, ThyroSeq® v3 in tailoring the often difficult clinical management of Hürthle cell thyroid nodules for optimal surgical treatment.

## 2. Case Presentation

**Figure 1:**
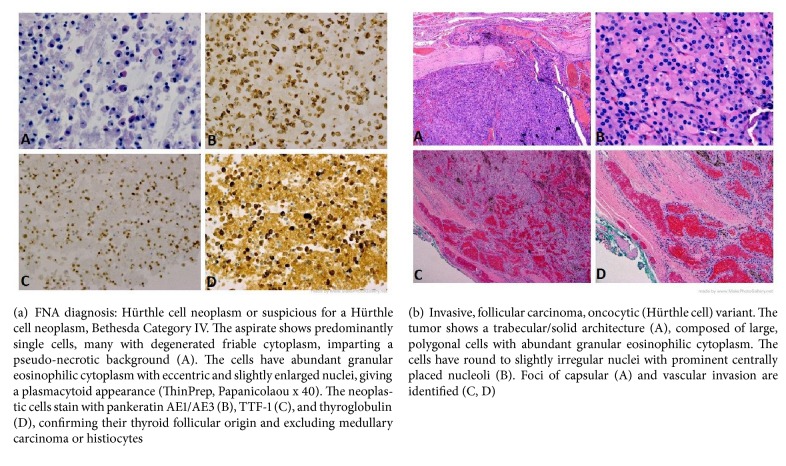


**Figure 2:**
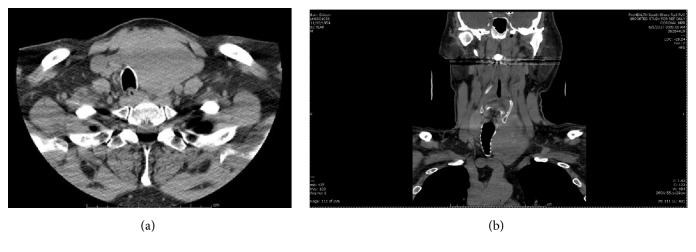
Preoperative neck CT without contrast, demonstrating a large left thyroid lobe mass with displacement of the trachea, mild compression, and early substernal extension; representative axial (a) and coronal views (b).

This patient was a generally healthy 62-year-old male with a left lobe complex nodule within a nontoxic multinodular goiter that had been enlarging for approximately 3 years. In 2015, the patient had a FNAB reported as benign, BC II. Because of continued growth, he had a second FNA biopsy approximately six months later reported as a Hürthle cell neoplasm or suspicious for a Hürthle cell neoplasm, BC IV with Oncocytic / Hürthle cells dispersed mostly singly and in small fragments in a background of lysed blood. CKAE1/AE3, TTF-1, and thyroglobulin immunostains were positive (Figure 1(a)). Molecular testing with ThyroSeq® v2 revealed an absence of gene mutations or fusions but overexpression of the MET gene with an uncertain increased risk of malignancy. After repeat ultrasound imaging, the nodule had grown from 4.9 to 6.0 cm over the course of 1 year. He was euthyroid with negative anti-thyroid antibodies. There was no family history of thyroid cancer or known radiation exposures in his youth. He had no obstructive symptoms despite the size of the mass and denied shortness of breath, dysphagia, neck pain, neck pressure, or recent voice changes. His weight had been stable and appetite good. His past medical history was significant for a retinal detachment, hypertension, and inguinal hernia with a surgical history limited to eye surgery and hernia repair. He denied tobacco or alcohol use. On exam, the patient had an enlarged, firm thyroid gland with the left thyroid lobe causing significant tracheal deviation to the right. A neck CT scan demonstrated a markedly enlarged left thyroid lobe (7.2 cm in sagittal height) causing significant rightward tracheal deviation, minimal tracheal compression, and slight early substernal extension (Figure 2). He had multiple opinions from both endocrinologists and surgeons with various recommendations from left thyroid lobectomy to total thyroidectomy. The patient had initially contemplated a hemithyroidectomy due to concerns for voice impairment that could impact his occupation as an attorney.

After a second surgical consultation, he elected to have another, more advanced molecular test performed on the same FNAB specimen. The ThyroSeq® v3 test has been designed to improve the performance of its previous version, ThyroSeq v2, specifically with respect to Hürthle cell tumors. This has been achieved by expanding the number of gene markers analyzed for mutations and gene fusions and particularly by incorporating the analysis of copy number alterations (CNAs), which are common in Hürthle cell cancers. ThyroSeq® v3 test results in this case showed CNAs involving multiple chromosomes with the pattern of genome haploidization which predicted a much greater probability that the left lobe nodule represented a Hürthle cell malignancy rather than Hürthle cell metaplasia or an adenoma. Based on the additional information provided by ThyroSeq® v3, in July, 2017, the patient elected a total thyroidectomy. At surgery, the overlying strap muscles were superficially adherent to the thyroid capsule on the left with a suspicion of minimal extrathyroidal extension of the tumor and a layer of muscle was left attached to the specimen. There were no paratracheal lymph nodes. He did require single gland parathyroid autotransplantation. Postoperatively, his parathyroid hormone and calcium levels were within normal limits. On final surgical pathology, an encapsulated 7 cm Hürthle cell carcinoma with 5 foci of angioinvasion was found along with foci of capsular invasion, without extrathyroidal extension (Figure 1(b)). A second opinion was sought and the reviewing pathologist reported 4 foci of capsular invasion and 3 foci of vascular invasion. The number of foci of vascular invasion was prognostically important and prompted more aggressive treatment and follow-up.

One month postoperatively, thyroglobulin was 308 ng/mL. A small thyroid remnant with 2.4% uptake in the surgical bed was found on I-131 whole body scan. An FDG-PET scan was negative for any activity in the thyroidectomy bed or for distant metastatic disease; therefore he was given 30 mCi of radioactive iodine to ablate the remnant. At this time, his thyroglobulin had decreased from 308 to 8.71 ng/mL. His postablation, I-131 whole body scan showed ablation of the thyroid remnant and no evidence of metastatic disease. By 10/27/2017, the thyroglobulin had decreased further to 0.2 ng/mL with no detectable thyroglobulin antibodies and a TSH of 0.09 uIU/ml indicating a favorable early response to initial treatment.

## 3. Discussion

In the past, cytologic assessment, with or without molecular profiling, of Hürthle cell nodules failed to accurately predict the risk of HCC. The presence of Hürthle or oncocytic cells in cytologic specimens from FNA samples is often seen in a wide range of thyroid pathologies, the majority of which are benign. The finding of predominance of Hürthle cells is usually interpreted as suspicious for a follicular neoplasm, Hürthle cell type, BC IV, conferring a positive predictive value (PPV) for malignancy of approximately 15-30%. The high frequency of nonneoplastic Hürthle cell proliferation in patients with Hashimoto's thyroiditis can be a diagnostic dilemma for the cytopathologist [3]. With the advent of molecular profiling, the hope was to minimize the need for diagnostic thyroid lobectomy for benign nodules, for tumor prognostication to tailor the extent of thyroid surgery for optimal cure and to prevent tumor recurrence. The Afirma® Gene Expression Classifier developed by Veracyte, Inc. (South San Francisco, CA) has been shown to have a high negative predictive value (NPV) for most benign thyroid nodules but with a poor PPV for malignancy and renders a large number of FNA samples with various proportions of nonneoplastic Hürthle cells as suspicious for malignancy, thus triaging most of these patients to thyroid surgery [4].

ThyroSeq is a multigene next-generation sequencing-based test for thyroid nodules. The early version, ThyroSeq v2, utilized the analysis of 56 genes predominantly for point mutations and gene fusions, as well as for limited gene expression alterations [2]. The expanded version of the test, ThyroSeq v3, interrogated 112 genes and is based not only on the analysis of point mutations, gene fusions, and gene expression alterations, but also on copy number alterations (CNAs) [5]. The latter is particularly important for predicting Hürthle cell carcinomas, which are known to have a characteristic pattern of CNAs with almost complete genome haploidization [6]. Taking advantage of the analysis of CNAs, in the validation study, ThyroSeq v3 showed reliable performance in Hürthle cell cancers, offering 93% sensitivity and 69% specificity [5]. In a preliminary report from a recent multicenter study which included 10 Hürthle cell carcinomas, 34 Hürthle cell adenomas, and 5 hyperplastic nodules with Hürthle cell predominance, the performance of ThyroSeq® v3 allowed for the detection of all HCCs (sensitivity, 100%; 95%CI: 69.2- 100%), with all 5 hyperplastic nodules with Hürthle cell predominance classified as negative and overall test specificity of 66.7% (95%CI: 49.8-80.9%) [7].

In an era of patient-guided decision-making and the ability to tailor the extent of surgery based on preoperative FNA biopsy prognostication, molecular profiling of thyroid nodules has become increasingly utilized. Despite the limitations of molecular testing and the variance in both PPV and NPV with a varying prevalence of malignancy in different populations, its utility in selecting patients for active surveillance, thyroid lobectomy, and total thyroidectomy will likely increase, especially as their overall accuracy improves over time. The particular advantages of ThyroSeq® v3 over ThyroSeq® v2 in guiding the extent of thyroid surgery for indeterminate Hürthle cell cytopathology are illustrated by this case report and helped tailor the best treatment for this patient with a Hürthle cell carcinoma who would otherwise have likely needed a completion thyroidectomy.
